# Cerebral Superficial Siderosis: Etiology, Neuroradiological Features and Clinical Findings

**DOI:** 10.1007/s00062-022-01231-5

**Published:** 2022-11-28

**Authors:** Stefan Weidauer, Elisabeth Neuhaus, Elke Hattingen

**Affiliations:** Institute of Neuroradiology (1), Goethe University, Schleusenweg 2–16, 60528 Frankfurt am Main, Germany

**Keywords:** Superficial siderosis, Infratentorial, Cortical, Amyloid related imaging abnormalities, Cerebral amyloid angiopathy

## Abstract

Superficial siderosis (SS) of the central nervous system constitutes linear hemosiderin deposits in the leptomeninges and the superficial layers of the cerebrum and the spinal cord. Infratentorial (i) SS is likely due to recurrent or continuous slight bleeding into the subarachnoid space. It is assumed that spinal dural pathologies often resulting in cerebrospinal fluid (CSF) leakage is the most important etiological group which causes iSS and detailed neuroradiological assessment of the spinal compartment is necessary. Further etiologies are neurosurgical interventions, trauma and arteriovenous malformations. Typical neurological manifestations of this classical type of iSS are slowly progressive sensorineural hearing impairment and cerebellar symptoms, such as ataxia, kinetic tremor, nystagmus and dysarthria. Beside iSS, a different type of SS restricted to the supratentorial compartment can be differentiated, i.e. cortical (c) SS, especially in older people often due to cerebral amyloid angiopathy (CAA). Clinical presentation of cSS includes transient focal neurological episodes or “amyloid spells”. In addition, spontaneous and amyloid beta immunotherapy-associated CAA-related inflammation may cause cSS, which is included in the hemorrhagic subgroup of amyloid-related imaging abnormalities (ARIA). Because a definitive diagnosis requires a brain biopsy, knowledge of neuroimaging features and clinical findings in CAA-related inflammation is essential. This review provides neuroradiological hallmarks of the two groups of SS and give an overview of neurological symptoms and differential diagnostic considerations.

## Introduction

**Fig. 1:**
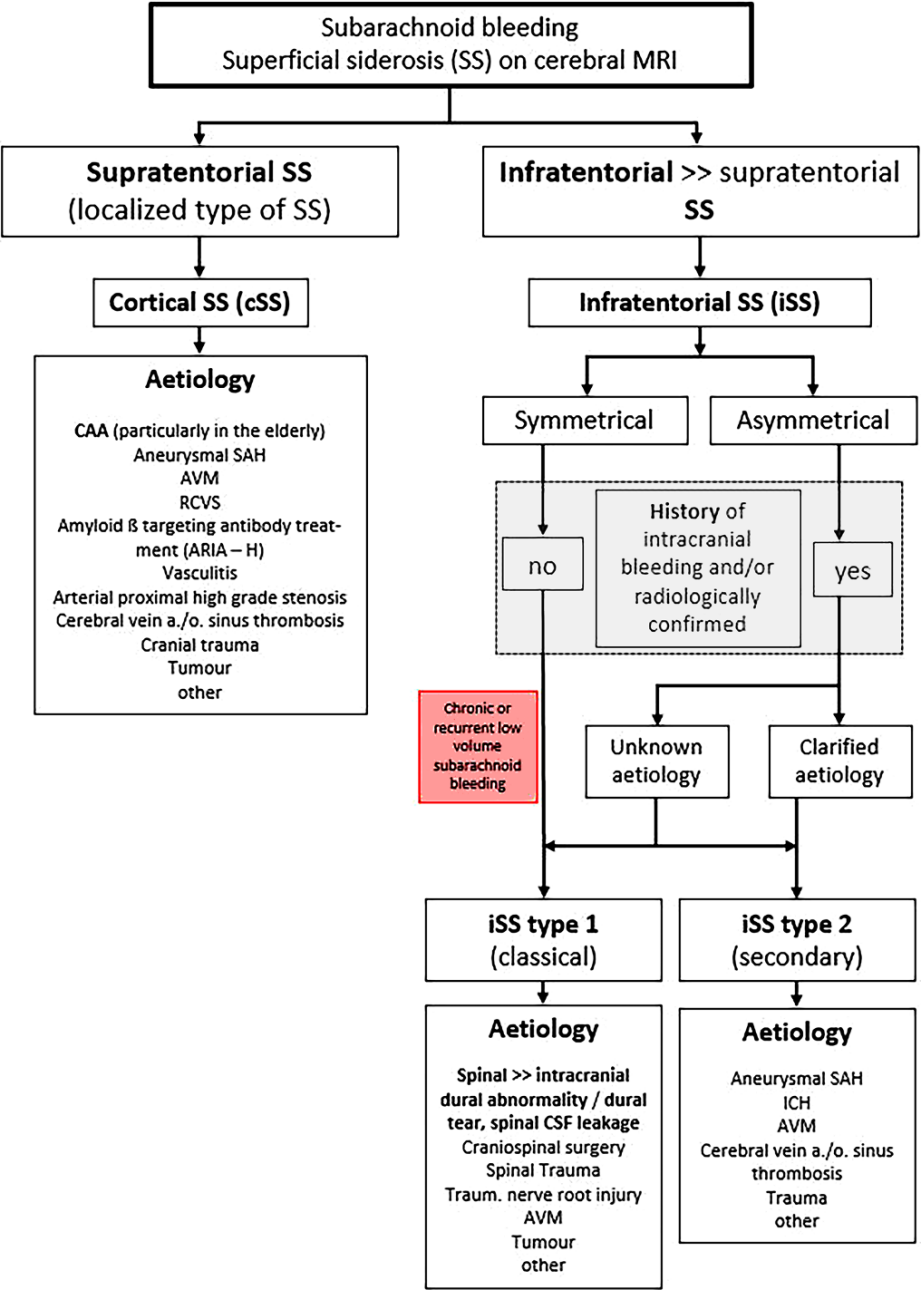
Algorithm of different types of superficial siderosis (SS) and the corresponding assumed etiologies. *ARIA—H*: amyloid-related imaging abnormalities, hemorrhagic type, *AVM* arteriovenous malformation, *CAA* cerebral amyloid angiopathy, *CSF* cerebrospinal fluid, *ICH* intracerebral hemorrhage, *RCVS* reversible cerebral vasoconstriction syndrome, *SAH* subarachnoid hemorrhage

Superficial siderosis (SS) of the central nervous system (CNS) constitutes linear hemosiderin in the leptomeninges and the superficial layers of the cerebral and cerebellar cortices, the brainstem and the spinal cord [1–3]. Infratentorial (i) SS was first described by Hamill in 1908 as a “case of melanosis of the brain, cord and meninges”, particularly involving the infratentorial structures in the posterior fossa and the spinal cord [4]. An iSS is often caused by chronic intermittent or continuous slight bleeding into the subarachnoid space [5–8]. The most common etiology is spinal dural abnormalities, often dural tears (classical or type 1 iSS). In addition, CNS tumors, arteriovenous malformations (AVM), head or spinal trauma and craniospinal surgery can also cause iSS [1, 2, 8–15]. Neuroimaging typically shows symmetrical involvement of posterior fossa structures. Less commonly, iSS may be due to an isolated causative subarachnoid hemorrhage (SAH) event, e.g. aneurysm rupture, AVM or CNS trauma. In this type 2 iSS (secondary iSS) magnetic resonance imaging (MRI) appearance of SS is likely asymmetric and predominantly focused around the bleeding site (see Fig. 1). In contrast to classical iSS, progressive cerebellar ataxia and impaired hearing are lacking on neurological examination [8, 15].

Besides iSS, a different type of so-called localized SS, i.e. cortical (c) SS can be differentiated, which is restricted to the supratentorial compartment (see Fig. 1; [15–25]). A cSS is characterized by asymmetric and focal areas of hemosiderin depositions in the cortical sulci, based on various etiologies other than in iSS [2, 9, 15–25]. Especially in older patients cSS is often due to cerebral amyloid angiopathy (CAA) [2, 18, 19]; however, not only the neuroradiological findings and the causes but also the clinical symptoms clearly differ between the two types of iSS [2, 9, 10, 15, 21]. This review deals with the characteristic neuroimaging features in iSS and cSS and also gives an overview of clinical symptoms and differential diagnostic considerations.

## Infratentorial Superficial Siderosis (iSS)

From a neuropathological point of view the hemosiderin deposits and especially the neurotoxic iron in the leptomeninges and the subpial structures lead to demyelination, axonal loss and subsequent atrophy [1, 3, 5, 6]. There is a sharp delineation of hemosiderin deposits in the cranial nerves and the spinal nerve roots directly at the transition zone of central glial cells and peripheral Schwann cells [1, 3, 5, 6, 13]. Therefore, the olfactory nerve and the vestibulocochlear nerve are preferentially involved, because both are pure glial nerves with close contact to the cerebrospinal fluid (CSF). Although the optic nerve represents the glial type, clinical signs of involvement are rare, possibly due to the shorter course through the subarachnoid space [7].

**Fig. 2:**
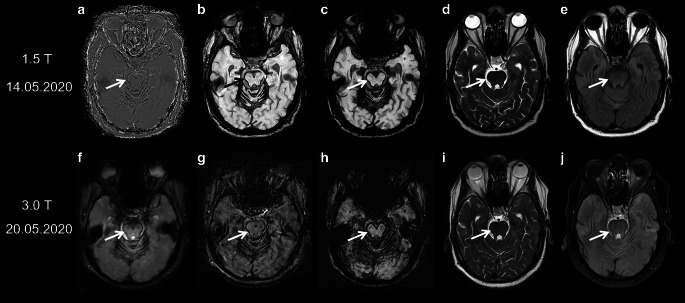
Diagnostic value of different MRI sequences in the detection of superficial siderosis (SS). A 61-year-old man with SS due to ongoing hemorrhage from a melanoma metastasis in the right frontal cortex. In T2*-GRE (**f**) and susceptibility-weighted imaging (SWI) (**b**,**g**), SS is revealed by dark rims on the surface of affected structures, e.g., the mesencephalon (*arrow*), with SWI being more sensitive. Minimum intensity projections (mIPs) of SW images (**c**,**h**) further enhance the conspicuousness of SS. In addition, filtered phase image of SWI (**a**) can be used to distinguish paramagnetic (hemorrhage/iron, *dark* here) from diamagnetic substances (calcification, *bright* here), as they have opposite signal intensities. In general, susceptibility effects are more pronounced on images acquired at 3 T (**f**–**j**) than on images acquired at 1.5 T (**a**–**e**). Whereas at 3 T a hypointense rim around the mesencephalon is seen in T2WI and fluid attenuated inversion recovery (FLAIR) images (**i**,**j**, *arrow*), SS is almost undetectable at 1.5 T in T2WI and FLAIR (**d**,**e**, *arrow*). **a–e** 1.5 T (Achieva dStream, Philips); **a**–**c** SWI, TR/α 52 ms/20°, 4 echoes TE1 = 12 ms, ∆TE = 11 ms; **d** T2, TR/TE/α = 5762 ms/110 ms/90°; **e** FLAIR, TR/TE/TI/α = 11000 ms/140 ms/2800 ms/90°; **f**–**j** 3 T (Skyra fit, Siemens); **f** T_2_*-GRE, TR/TE/α = 631 ms/20 ms/20°; **g**, **h** SWI, TR/TE/α = 27 ms/20 ms/15°; **i** T2, TR/TE/α = 4980 ms/92 ms/150°, **j** FLAIR: TR/TE/TI/α = 8500 ms/81 ms/2440 ms/150°. *TR* repetition time, *TE* echo time, *TI* inversion time, *α* flip angle

For many years a definitive diagnosis of SS could be established only by biopsy or post-mortem; however, due to the iron sensitive T2* gradient recalled echo (GRE) sequences or the more sensitive susceptibility weighted imaging (SWI) especially at higher field strength, SS exhibits characteristic imaging features with signal loss, i.e. dark rims on the surface of the affected structures [2, 18, 26–31]. The paramagnetic blood breakdown products, also including hemosiderin as a stable final product, cause local magnetic field inhomogeneity [2, 30, 31]. The radiological appearances of SS with different sequences and field strengths are illustrated in Fig. 2.

**Fig. 3:**
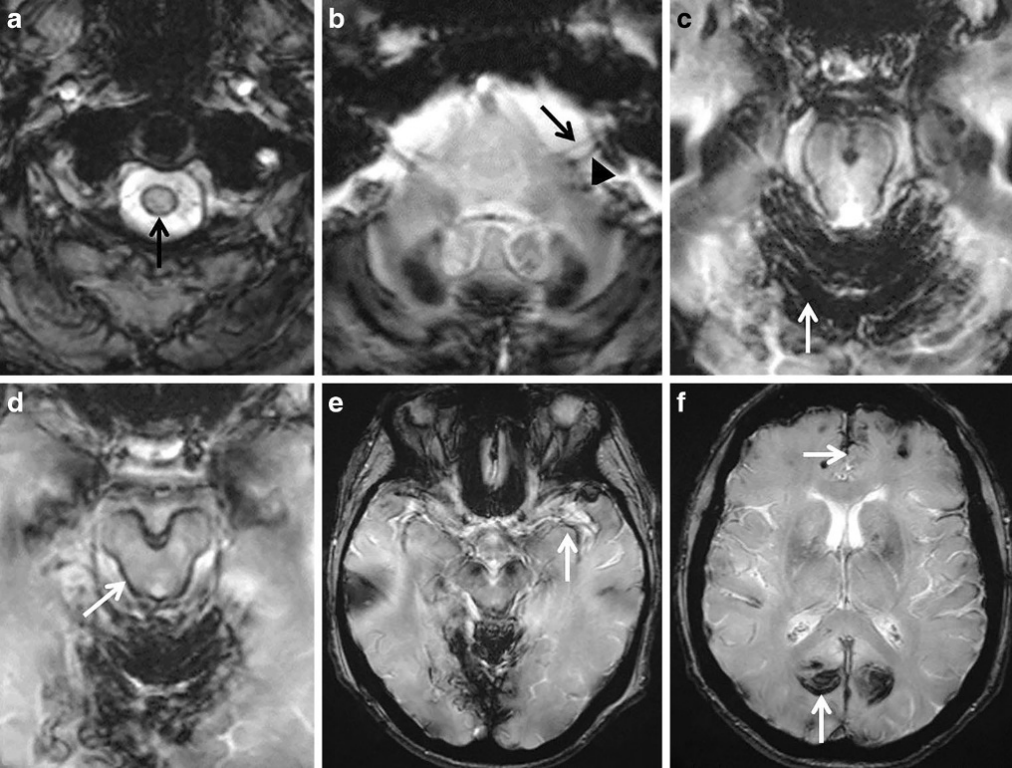
Classical infratentorial superficial siderosis (iSS) in a 77-year-old woman with progressive hearing loss, gait ataxia, visual disturbances and optical hallucinations over 6 months. Axial T2*-GRE (**a**–**f**) showing SS of the upper cervical spinal cord (**a**, *arrow*), the VIII cranial nerve but sparing the VII cranial nerve (**b**: a*rrowhead*, *arrow*), the cerebellum and mesencephalon (**c**,**d**: *arrow*), the medial Sylvian fissure (**e**, *arrow*) and of the medial occipital lobes (**f**, *arrow*)

**Fig. 4:**
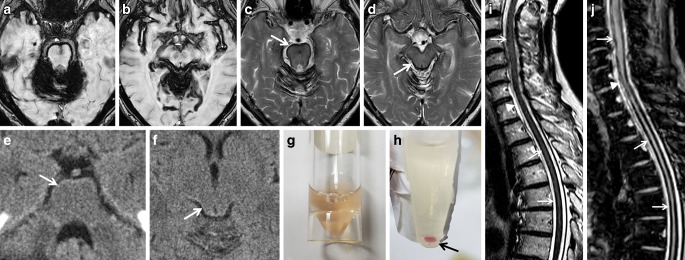
A 73-year-old woman suffering from recurrent severe headache attacks due to ventral dural defect at the level of the second thoracic vertebra with spontaneous intracranial hypotension and recurrent subarachnoid bleeding over more than 10 years. Axial susceptibility-weighted imaging (SWI) (**a**,**b**) and axial T2-weighted images (WI) (**c**,**d**; *arrow*) showing extensive superficial siderosis (SS) especially infratentorial; **e**, **f** CT disclosing slight hyperdense pontine and mesencephalic surface (*arrow*). Cerebrospinal fluid (CSF) analysis demonstrating auburn liquor (**g**); **h** xanthochrome supernatant and sedimentation of erythrocytes after centrifugation (**h**, *arrow*). **i**,**j** (SWI sag.) Extensive spinal SS (*arrows*) and ventral epidural fluid collection at the upper thoracic level (*arrowheads*)

**Fig. 5:**
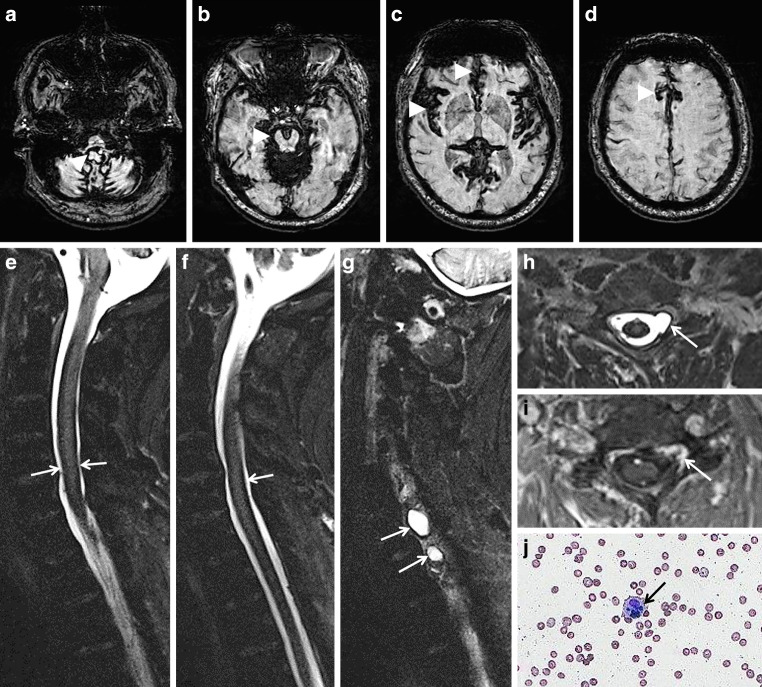
Classical superficial siderosis (SS) in a 59-year-old man suffering from progressive gait ataxia within 6 months and traumatic nerve root injury C7 and C8 30 years ago. **a**–**d** Axial susceptibility-weighted imaging (SWI) showing SS with pial signal loss (*arrowheads*) especially in the posterior fossa (**a**,**b**) and partially supratentorial (**c**,**d**: *arrowheads*). **e**–**j** SS also of the spinal cord (**e**,**f**: T2*-weighted images [WI] sag.; *arrows*); enlarged empty nerve root pouches C7 and C8 left (**g**,**h**: T2* WI sag. and ax. *arrows*) with inhomogeneous contrast enhancement (**i**, post contrast T1 WI ax. *arrow*); **j** cerebrospinal fluid (CSF) analysis exhibits silent chronic subarachnoid bleeding with erythrocytes and siderophages (*arrow*; magnification: 200x)

The most common etiology is spinal dural disease, often coming along with ventral dural tears, less commonly intracranial dural abnormalities inducing classical type of iSS (type 1) (see Figs. 3 and 4). Dural tears may be caused by at times calcified disc herniation and occasionally spiculated osteophytes, often associated with a ventrally accentuated epidural fluid collection due to CSF leakage [10–12, 14, 15]. Dural ventral tears are preferentially located in the upper thoracic spinal levels [8, 14, 15]. Further pathologies are intrinsic dural diseases caused by connective tissue abnormalities, spinal CSF venous fistula or nerve root diverticula, traumatic nerve root avulsion (see Fig. 5) and postoperative pseudo-meningoceles [8, 14, 15, 32]. Spontaneous intracranial hypotension (SIH) due to CSF leakage with similar intraspinal epidural fluid collection is associated with leptomeningeal hemosiderosis on MRI in 5–10% of patients [14, 33, 34]. Other less common etiologies for classical iSS are neurosurgical craniospinal interventions, trauma, cranial or spinal tumors and AVM [1, 8, 15]. Chronic ongoing or repetitive low-volume bleeding in the subarachnoid space can occur before the diagnostic conformation of a CNS tumor (see Fig. 2; [11]) or may be due to postoperative residual tumor tissue or a postsurgical cavity. There is evidence that AVMs found in the diagnostic work-up of SS are often incidental [8].

In type 2 iSS (secondary iSS) [5–13, 15] evidence of a causative often single SAH or parenchymal bleeding is radiologically present; however, in contrast to classical iSS MRI may disclose asymmetric iSS predominantly focused in the neighborhood of the bleeding site [8, 15]. It is worth noting that parenchymal bleeding may be caused by a venous outflow disorder. In rare cases venous thrombosis can occur as a result of decreased intracranial pressure in so far undetected spinal dural CSF leakage [10, 12]. Therefore, it seems recommendable that the etiology of the bleeding event has to be clarified before the assignment to type 1 or 2 iSS is made (see Fig. 1).

**Fig. 6:**
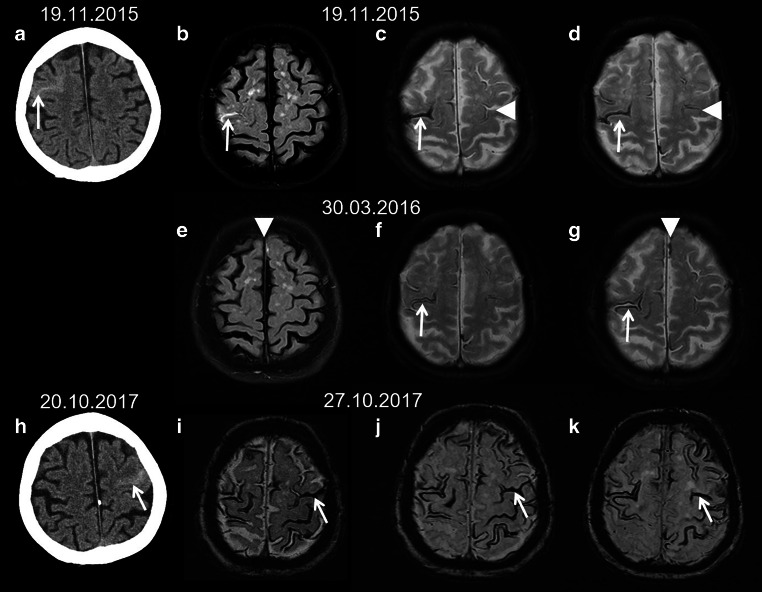
A 74-year-old woman suffering from recurrent cortical subarachnoid hemorrhage (cSAH) in cerebral amyloid angiopathy (CAA). **a**–**d** First cSAH frontal right (**a**: CT ax., *arrow*) with sulcal hyperintense signal changes on fluid attenuated inversion recovery (FLAIR) images (**b**, *arrow*) and sulcal signal loss on T2*WI (**c**,**d**: *arrow*); additional cortical superficial siderosis (cSS) left (**c**,**d**: *arrowhead*); **e**–**g** second cSAH paramedian frontal left (**e**: FLAIR ax.; **g**: T2*WI ax.; *arrowhead*); note characteristic bilinear track-line appearance of cSS in the chronic stage (**f**,**g**: T2*WI, *arrow*); **h**–**k** third cSAH frontodorsal left (**h**: CT ax., *arrow*) with signal loss on SWI (**i**–**k**, *arrow*) and progressive cSS bilaterally

**Fig. 7:**
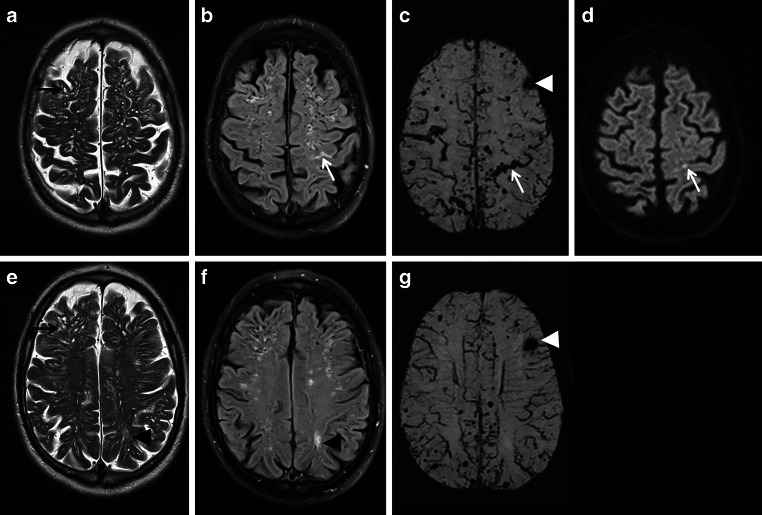
A 72-year-old man with beginning dementia suffering from temporary hemiparesis right and aphasia due to cerebral amyloid angiopathy (CAA). Acute cortical subarachnoid hemorrhage (cSAH); **b** fluid attenuated inversion recovery [FLAIR ax.; **c** susceptibility-weighted imaging (SWI) ax.; **d** diffusion-weighted imaging (DWI) ax., b = 1000 s/mm^2^, *arrow*. Enlarged perivascular spaces (PVS) (**a**,**e**: T2WI ax.; *arrow*), focal small gliosis (**b**,**f**: FLAIR ax.; *arrowhead*), multiple microbleeds (MB) and cortical superficial siderosis (cSS) (**c**,**g**: SWI ax.) beside residual atypical intracerebral bleeding frontal left (**c**,**g**: *arrowhead*)

**Fig. 8:**
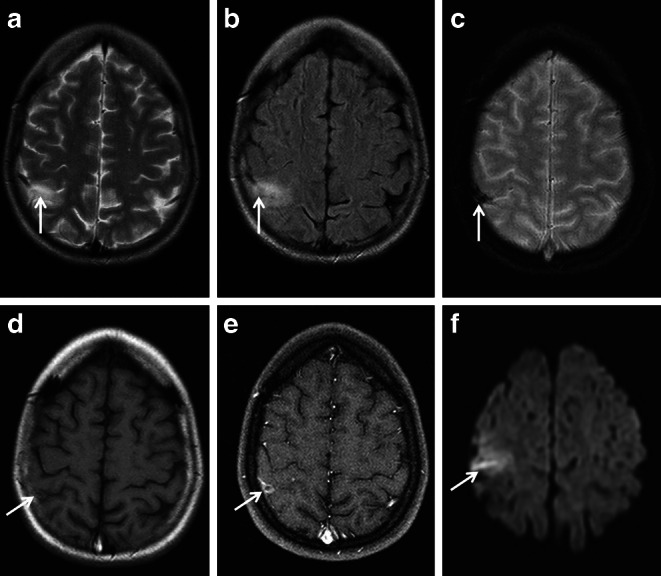
Cortical vein thrombosis as a possible mimic of cortical superficial siderosis (cSS) in a 27-year-old woman with right-sided headache and sensory Jacksonian seizures. MRI demonstrating cortical hyperintense lesion postcentral parietal right (**a**: T2 WI ax.; **b** fluid attenuated inversion recovery [FLAIR] images ax.; *arrow*), signal loss and “blooming” of the central vein (**c**, T2*WI ax.; *arrow*) without cSS, circumscribed peripheral contrast enhancement (**d**,**e** T1 WI ax., post contrast T1 WI ax.; *arrow*) and restricted diffusion of the thrombus (**f**, diffusion-weighted imaging [DWI] ax., b = 1000 s/mm^2^, *arrow*)

**Fig. 9:**
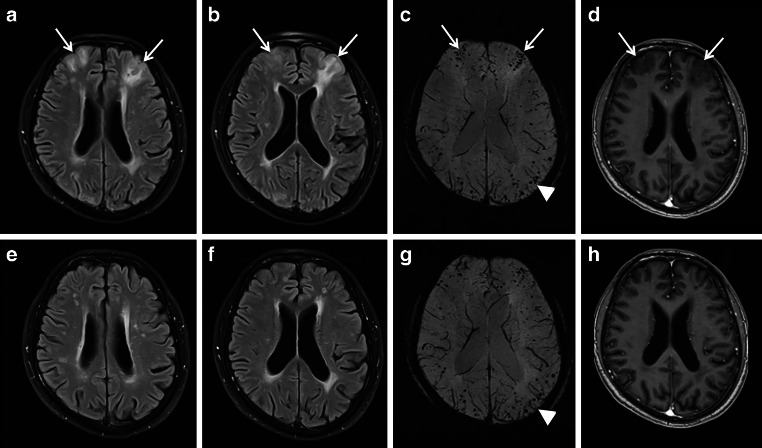
CAA related inflammation (CAA-ri) in a 61-year-old man suffering from subacute psychosyndrome with disturbance of consciousness and executive disorders. **a**–**d** bifrontal left dominant hyperintense lesions with sulcal effusions (**a**,**b**: fluid attenuated inversion recovery [FLAIR] images ax.; *arrows*), accentuated microbleeds (MB) (**c**, susceptibility-weighted imaging [SWI] ax.; *arrows*) and hypointense signal conversion on postcontrast (pc) T1 WI (**d**, *arrows*) with enhancement; **e**–**h** follow-up MRI 13 months later after 3 bouts of high-dose methylprednisolone infusions, neurological examination was unremarkable. Completely resolved lesions frontal (**e**,**f**), unchanged cortical and subcortical MB (**c**,**g**; *arrowheads*)

Typical neurological manifestations of classical iSS (type 1) are slowly progressive sensorineural hearing impairment and cerebellar symptoms, such as ataxia, kinetic tremor, nystagmus and dysarthria (see Figs. 3 and 4; [7–9, 13]). Preferential affection of the cerebellar vermis results in severe ataxic gait disturbance up to inability to stand and walk [8]. In addition, spinal cord symptoms may occur, especially corticospinal tract signs with spasticity, rarely also anterior horn signs (see Figs. 4 and 5; [6, 8, 12]). In contrast, in patients suffering from iSS type 2 these neurological symptoms are lacking [8, 15]. Contrariwise, predominately focal neurological deficits are often present depending on the localization and etiology of the pathologic process.

Patients suffering from SIH as sequelae of spinal dural CSF leakage often show orthostatic headache, dizziness and auditory disturbance, nausea and vomiting. Impressive amnestic hint is the statement “the day it all began” [10, 12, 14, 34]. Characteristic focal neurological symptoms are cranial nerve palsies, especially abducens nerve failure [14, 34]. Brain sagging results in consecutive mechanical stress of the abducens nerve due to the fixation within Dorello’s canal when entering the clivus [34].

Beside MRI with thin slices, e.g. constructive interference in steady-state (CISS) and 3D T2 sampling perfection with application optimized contrasts using different flip angle evolutions (SPACE) [35], myelographic computed tomography (CT) and especially if indicated dynamic subtraction myelography are necessary to identify the circumscribed dural defect in classical iSS (see Fig. 1; [14, 36–38]). Overall, in more than 80% of patients with iSS a potentially causal spinal or cranial dural abnormality can be identified [8, 10, 15, 35]. An additional supportive therapeutic option in iSS is the administration of iron chelates [39].

## Cortical Superficial Siderosis (cSS)

A cSS is a sequela of a previous acute cortical (c; or convexity) SAH with focal hyperdense sulcus on CT and hyperintense sulcal signal changes on fluid attenuated inversion recovery (FLAIR) images (see Fig. 6; [2, 16–19]). Terminology also includes subarachnoid hemosiderosis, sulcal siderosis and superficial cortical siderosis [2]. There is a further differentiation between local cSS involving 1–3 sulci and disseminated cSS affecting at least 4 sulci [40]. Whereas in the acute or subacute stage T2* WI and SWI show often homogeneous signal loss, in the chronic stage bilinear track-like appearance is typical (see Fig. 6; [2, 18]). Especially in older patients cSS is often due to cerebral amyloid angiopathy (CAA) (see Fig. 7). Further etiologies of cSS are aneurysm related SAH, AVM, reversible cerebral vasoconstriction syndrome (RCVS), vasculitis, arterial proximal high-grade stenosis, cerebral vein and/or sinus thrombosis, cranial trauma and also amyloid beta targeting antibody treatment (see Fig. 1; [8, 15, 41–49]).

### Cerebral Amyloid Angiopathy (CAA)

CAA encompasses a genetic and biochemical inhomogeneous group of pathologies in which the reduced perivascular clearance of amyloid beta (Aß) from the interstitial fluid has a key role in the pathogenesis of CAA and Alzheimerʼs disease (AD) [50–55]. Beside an impairment of the intramural periarterial drainage (IPAD) an insufficient perivascular transport via the glymphatic system is also discussed [54–56]. In consequence, there are deposits especially in the small and medium sized arteries in the cortex and the leptomeninges with preference of the posterior lobar brain regions [54, 57–59]. Whereas capillary involvement is classified as CAA type 1, type 2 reflects CAA without deposits in the capillaries [60–63]. Apolipoprotein (APO) E ɛ4 expression is a risk factor for CAA especially with capillary type 1 and APO E ɛ2 is associated with type 2 [60–63]. Aß-42 is less soluble and parenchymal fibrils are a likely consequence, while the more soluble Aß-40 preferentially accumulates in the vessel walls [60, 64–66]. Arterial pulsation and vasomotion generated by the smooth muscle cells enable and facilitate interstitial drainage [53–55]; however, vascular Aß deposits interfere with these mechanisms due to reduced vessel wall volubility, establishing a self-reinforcing cycle of reduced Aß clearance and widened perivascular spaces (see Fig. 7; [54, 60, 67, 68]).

Neuroradiological hallmarks of CAA are multiple cortical and subcortical lobar microbleeds (MB) [27, 60, 69]. In contrast, MB caused by lipohyalinosis, arteriosclerosis and fibrinoid necrosis of the small perforators related to aging and common vascular risk factors, i.e. arterial hypertension and diabetes, are located in the basal ganglia, thalamus, pons and the cerebellum [27, 70–77]. Consecutively, typical intracerebral hemorrhages (ICH) associated with hypertension appear in these regions, whereas CAA related atypical ICH are located in the cerebral lobes with high risk of recurrence (see Fig. 7; [69, 70, 78–81]). APO Eɛ2 is a risk factor for hemorrhagic CAA, whereas the APO Eɛ4 allele is a major risk factor for AD and CAA, the latter often with a severe clinical course [60, 64, 82–86]. In addition, CAA induce white matter hyperintensities with conflating appearance over time (see Fig. 7; [60, 73, 75, 77]). As a result of interaction between neurodegenerative and cerebrovascular processes in cerebral Aß deposition, subcortical MB preferentially parieto-occipital not only occur in CAA but also in AD [60, 73, 77, 87–92].

Although cSS includes several etiologies of cSAH (see Fig. 1; [2, 8, 15, 41–49]), especially in older individuals cSS is an important neuroimaging feature in CAA [2, 17, 19, 25, 60, 69, 93]. In the seminal publication by Linn et al. in 2010 [19] cSS was detected in 60.5% of patients suffering from CAA, mean age 70 ± 6.4 years. In contrast none of the controls showed cSS. Whereas the classic Boston criteria had a sensitivity of nearly 90% for CAA related hemorrhage, inclusion of cSS raised the sensitivity up to 94.7% [19]. In consequence, focal or disseminated cSS beside singular lobar cortical or subcortical hemorrhage were included as imaging criteria for probable CAA in the modified Boston criteria (see Fig. 6; [69, 70]).

Typical neurological presentation of cSS in CAA includes transient focal neurological episodes or “amyloid spells” [9, 83, 94–96]. These represent stereotypical positive or negative neurological symptoms depending on the localization of the initial cSAH and the developing cSS. For example, involvement of the central sulcus with affection of the precentral or postcentral gyrus will cause contralateral propagating sensory or motor symptoms. From a pathophysiological point of view cortical spreading depolarization is discussed [83, 94, 96, 97]. Knowledge of this clinical feature in CAA associated with cSS is crucial. Focal epileptic seizures, e.g. sensory or motor Jacksonian seizures or ischemia, e.g. transient ischemic attacks, may mimic cSS related symptoms with possible wrong therapeutic consequences of antiepileptic or antithrombotic medication [75, 77, 95, 96]. Further differential diagnoses include focal vasospasms, RCVS and cortical venous thrombosis (see Fig. 8; [15, 41, 42, 73]). Neuroradiological hints and clinical symptoms to differentiate imaging mimics of cSS from “true” cSS are summarized in Table 1 [30, 31, 41, 42, 98–102].

**Table 1:** Mimics of cortical superficial siderosis

Disease	Differential diagnostic hints	
	Imaging features	Neurological symptoms
Cortical vein thrombosis	*MRI*	–
	Pronounced “blooming effect” (T2*WI, SWI)	Focal epileptic seizures (e.g. sensible or motor Jacksonian seizures)
	Tubular aspect (parallel to thrombosed vein)	Focal neurological deficits
	Facultative hyperintense signal (T2, FLAIR) of adjacent cortex	(Facultative progressive, prolonged onset)
	Facultative intravasal diffusion restriction	Facultative headache
	*CT*	–
	“Cord sign” (hyperdense vein sign)	–
	“Missing vein”, filling defect (CTA)	
Cortical hemorrhagic transformation in cerebral infarcts	*MRI*	–
	Petechial or broad linear or serpiginous signal loss (T2*WI, SWI)	Apoplectiform onset of (focal) neurological deficits
	Often additional subcortical tissue damage	Focal (or secondary generalized) seizures
	Acute/subacute stage: diffusion restriction	Facultative headache
	Chronic stage: gliosis	
	*CT*	–
	Hyperintense cortical band	–
	Often hypointense additional cerebral infarct	
Laminar cortical necrosis (e.g. hypoxic injury, status epilepticus)	*MRI*	–
	T1WI: hyperintense cortical/bandlike signal	Focal neurological deficits
	T2*WI/SWI: hypointense cortical/bandlike signal	Seizures
	DWI: cortical/band-like diffusion restriction in the acute stage	Facultative impairment of consciousness
		Facultative disturbance of vigilance
		Different states of confusion
Sturge Weber syndrome	*MRI*	–
	T2*WI/SWI: hypointense signal/signal loss possibly cortical and linear	Phacomatosis (encephalotrigeminal angiomatosis)
	SWI phase: negative (differentiation between diamagnetic mineralization and paramagnetic hemosiderin)	Neuropsychological deficits
		Seizures
	*CT*	–
	Hyperdense possibly bandlike calcifications	–
Calcifying angiopathy/mineralizing microangiopathy	*MRI*	–
	T2*WI/SWI: cortical bandlike, linear hypointense/signal loss, often symmetric	Slowly progressive focal neurological symptoms, e.g. visual disturbances
	SWI phase: negative	
	Especially occipital lobes	
	*CT*	–
	Bandlike hyperdense cortex, often symmetric	–
Cockayne syndrome	*MRI*	–
	T2*WI/SWI: hypointense signal/signal loss in the basal ganglia, less often in the dentate nucleus and cortex	Neurodegenerative disorder
	SWI phase: negative	Four clinical overlapping syndromes
	Myelination disorder (hypomyelination or demyelination)	Congenital cataract
	Major brain atrophy	Type 1 (classical type) begins in infancy, death occurs in first decades of life
	*CT*	–
	Calcification	–

*CT* Computed Tomography, *CTA* Computed tomography angiography, *FLAIR* Fluid attenuated inversion recovery, *MRI* Magnetic resonance imaging, *SWI* Susceptibility-weighted imaging, T2*WI T2*-weighted images

### CAA Related Inflammation (CAA-ri)

CAA related inflammation (CAA-ri) is a disease subtype associated with autoantibodies against Aß deposits in the vessel walls of cortical and leptomeningeal small and medium sized arteries, arterioles and capillaries [103–106]. The vascular and perivascular inflammation cause vasogenic edema and sulcal effusions with hyperintense signal changes on T2 WI and FLAIR images, i.e. amyloid related imaging abnormalities-edema (ARIA-E) (see Fig. 9; [48, 49, 107, 108]). The hemorrhagic type (ARIA-H) shows cerebral MB and cSS [40, 41, 108]. Neurological presentation of CAA-ri is characterized by rapidly progressive cognitive decline with impairment of consciousness, headache, seizures and variable focal neurological deficits depending on the localization of the autoimmune process [104, 109–111]. Diagnostic criteria differentiate between probable and possible CAA-ri [104]. In probable CAA-ri MRI discloses uni- or multifocal subcortical or deep white matter hyperintensities that are asymmetric and extend to the immediately subcortical white matter, and asymmetry is not due to past ICH [48, 104, 105, 110]. The patients are of age ≥ 40 years and neoplastic, infectious or other etiologies must be excluded. Because definitive diagnosis requires brain biopsy, knowledge of neuroradiological features in CAA-ri is essential [104, 110, 112]; however, from a histological point of view CAA-ri summarizes perivascular inflammation with histiocytes and also vessel wall inflammation with lymphocytes, and changeover to Aß related angiitis (ABRA) is not further differentiated [113–116].

There is evidence that intravenous (i.v.) high-dose corticosteroid pulse therapy with slow oral tapering is effective in spontaneous CAA-ri with neurological recovery in 84% within 1 year (see Fig. 8; [104, 110, 117, 118]); however, especially when i.v. corticosteroid therapy is stopped suddenly, in 34% recurrence within 24 months was observed. Focal brain atrophy is a likely consequence in nonresponders to anti-inflammatory treatment [104, 112].

### Amyloid beta (Aß) Targeting Monoclonal Antibody Therapies

Different randomized clinical trials within the investigational use of monoclonal antibodies targeting Aß including aducanumab and bapineuzumab showed ARIA‑E and ARIA‑H. This suggests that immunotherapy related ARIA is an iatrogenic version of CAA-ri [40, 59, 81, 82, 108, 119, 120]. Due to increased parenchymal trafficking of Aß to the perivascular pathway during immunization with monoclonal antibodies the Aß overflow may lead to a disruption of smooth cells in the vessel wall [54, 60]. The extravasation of fluid with elevated protein content causes ARIA‑E with edema and sulcal effusions, depending on the location of affected intraparenchymal and/or leptomeningeal vessels (Fig. 9; [40, 48, 60, 81, 82]). Whereas a single hyperintense lesion on FLAIR images smaller than 5 cm reflects mild severity, lesions > 5 and ≤ 10 cm are classified as moderate and lesions > 10 cm reflect severe ARIA‑E [48]. Extravasation of blood cells causes ARIA‑H, whereas up to 4 MB are considered as mild, 5–9 MB reflects moderate and ≥ 10 MB reflects severe ARIA‑H [50]. In addition, also new areas of cSS (1, 2 or > 2) represent a mild, moderate or severe stage, respectively [40, 81]. The number of MB at baseline and the APO-Eɛ4 allele are risk factors for ARIA‑E and ARIA‑H. The risk of ARIA‑E also depends on the antibody dosage and patients suffering from ARIA‑E are at higher risk for additional ARIA‑H [40, 108]. In the EMERGE and ENGAGE phase 3 randomized clinical trials of aducanumab, ARIA‑H associated cSS occurred in total in 14.7% of patients treated with a dose of 10 mg/kg and in APO-Eɛ4 carriers in 19.1% [108].

**Fig. 10:**
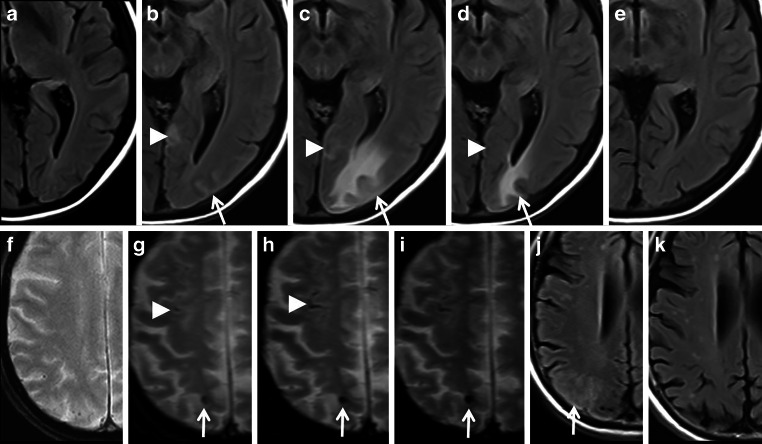
Amyloid related imaging abnormalities (ARIA). **a**–**e** fluid attenuated inversion recovery [FLAIR] images ax. showing encephalopathic type of ARIA (ARIA—E) in a 54-year-old man treated with aducanumab, weeks 14 (**a**), 30 (**b**), 34 (**c**), 38 (**d**) 40 (**e**) after treatment initiation; sulcal effusions (**b**–**d**, *arrowhead*) and additional hyperintense lesion in the occipital lobe (**b**–**d**, *arrow*), which completely resolved at week 40. T2* WI ax. (**f**–**i**) and FLAIR ax. (**j**,**k**) demonstrating hemorrhagic type of ARIA (ARIA-H) and ARIA‑E in a 68-year-old woman treated with aducanumab at baseline (**f**), weeks 14 (**g**), 18 (**h**,**j**), 20 (**i**) and 94 (**k**)

However, it is noteworthy that despite possible impressive imaging features the most common associated neurological symptom was headache [108]. Whereas ARIA‑E was transient and resolved within 12–16 weeks after initial detection, ARIA‑H tends to persist over time (see Fig. 10). It is hypothesized that vascular remodelling after Aß clearance might reduce further risk of ARIA over time [60, 108].

In conclusion, iSS is likely due to recurrent or continuous slight bleeding into the subarachnoid space, commonly due to spinal dural abnormalities, often dural tears (classical or type 1 iSS). Dural tears may be caused by at times calcified disc herniation and occasionally spiculated osteophytes, often associated with a ventrally accentuated epidural fluid collection due to CSF leakage. Further pathologies are intrinsic dural diseases caused by connective tissue abnormalities, CSF-venous fistula or nerve root diverticula, traumatic nerve root avulsion and postoperative pseudo-meningoceles. In consequence, detailed neuroradiological assessment of the spinal compartment is necessary, including MRI with thin slices, e.g. CISS and SPACE sequences, myelographic computed tomography (CT) and dynamic subtraction myelography. In SIH due to CSF leakage with similar intraspinal epidural fluid collection MRI concomitantly disclosed leptomeningeal hemosiderosis in 5–10% of patients.

In contrast, cSS especially in older patients is often due to CAA, encompassing a genetic and biochemical inhomogeneous group of pathologies in which the reduced perivascular clearance of Aß from the interstitial fluid has a key role in the pathogenesis. Typical clinical presentation of cSS in CAA includes transient focal neurological episodes or “amyloid spells”. Knowledge of this neurological feature in CAA and associated cSS is essential to avoid clinical misinterpretation and subsequent wrong therapeutic interventions. In addition, CAA-ri may occur spontaneously or caused by Aß immunotherapy. In contrast to several grades of neuropsychological disturbances due to spontaneous CAA-ri, Aß immunotherapy associated ARIA‑E and ARIA‑H neurologically is often present with headache. In contrast, slowly progressive sensorineural hearing impairment and cerebellar symptoms up to severe ataxic gait disturbance reflect the neurological key symptoms in the classical type of iSS.
